# Hybrid Deep Learning Approach for Stress Detection Using Decomposed EEG Signals

**DOI:** 10.3390/diagnostics13111936

**Published:** 2023-06-01

**Authors:** Bishwajit Roy, Lokesh Malviya, Radhikesh Kumar, Sandip Mal, Amrendra Kumar, Tanmay Bhowmik, Jong Wan Hu

**Affiliations:** 1 Department of Computer Science Engineering-AI & ML, Siliguri Institute of Technology, Siliguri 734009, India; bishwamail@gmail.com; 2 School of Computing Science and Engineering, Vellore Institute of Technology Bhopal University, Bhopal 466114, India; lokesh.malviya2020@vitbhopal.ac.in (L.M.); sandip.mal@vitbhopal.ac.in (S.M.); 3 Department of Computer Science and Engineering, National Institute of Technology, Patna 800001, India; radhikesh12@gmail.com; 4 Department of Civil Engineering, Roorkee Institute of Technology, Roorkee 247667, India; amrendraroy2k8@gmail.com; 5 Department of Computer Science and Engineering, Pandit Deendayal Energy University, Gandhinagar 382426, India; tanmaybhowmik@gmail.com; 6 Department of Civil and Environmental Engineering, Incheon National University, Incheon 22022, Republic of Korea; 7 Incheon Disaster Prevention Research Center, Incheon National University, Incheon 22022, Republic of Korea

**Keywords:** EEG, DWT, CNN, LSTM, BiLSTM, GRU

## Abstract

Stress has an impact, not only on a person’s physical health, but also on the ability to perform at the workplace in daily life. The well-established relation between psychological stress and its pathogeneses highlights the need for detecting psychological stress early, in order to prevent disease advancement and to save human lives. Electroencephalography (EEG) signal recording tools are widely used to collect these psychological signals/brain rhythms in the form of electric waves. The aim of the current research was to apply automatic feature extraction to decomposed multichannel EEG recordings, in order to efficiently detect psychological stress. The traditional deep learning techniques, namely the convolution neural network (CNN), long short-term memory (LSTM), bidirectional long short-term memory (BiLSTM), gated recurrent unit (GRU) and recurrent neural network (RNN) models, have been frequently used for stress detection. A hybrid combination of these techniques may provide improved performance, and can handle long-term dependencies in non-linear brain signals. Therefore, this study proposed an integration of deep learning models, called DWT-based CNN, BiLSTM, and two layers of a GRU network, to extract features and classify stress levels. Discrete wavelet transform (DWT) analysis was used to remove the non-linearity and non-stationarity from multi-channel (14 channel) EEG recordings, and to decompose them into different frequency bands. The decomposed signals were utilized for automatic feature extraction using the CNN, and the stress levels were classified using BiLSTM and two layers of GRU. This study compared five combinations of the CNN, LSTM, BiLSTM, GRU and RNN models with the proposed model. The proposed hybrid model performed better in classification accuracy compared to the other models. Therefore, hybrid combinations are appropriate for the clinical intervention and prevention of mental and physical problems.

## 1. Introduction

Human life today is not as simple as it once was. According to a recent study, the greatest impact on routine life is on working professionals aged 25 to 40 [1]. Stress has been revealed to be a silent killer for the human brain. Stress is the root cause of every mental problem, and is due to various physical or emotional states in the human body. Work pressures to meet deadlines, financial crunches, job dismissals, unemployment and various corporate demands contribute to increasing stress levels. The human brain progresses to stressors in order to maintain balance in the nervous system [1]. If professionals are under stress for an extended period, their performance suffers, and they become distressed. This stress seems to have negative impacts on the human body, causing diseases such as insomnia, decreased immunity, infections, cervical impairments and migraines, and so on [1]. A person is unaware of the stress that is silently killing his/her mind. Scenario and strategic management from health care institutions, digital technologies for stress prediction and entrepreneurship development on the national medical services market could boost a population’s quality of life and its nation’s human potential [2,3,4].

The main entities that reflect stress are the following: human body temperatures, brain activity and eye blinks [1]. As stated by a World Health Organization (WHO) survey, depression is one of the leading causes of disability worldwide [5]. In accordance with India’s national mental health survey (2015–2016), held by the National Institute of Mental Health and Neuro Sciences (NIMHANS) [2], the population diagnosed with mental illness has increased from 7.5 percent in 2014 to 10.6 percent in 2016. The ratio of patients with doctors in the low- and middle-income classes is being threatened [5]. In India, more than 150 million people are suffering from different mental illnesses such as anxiety, depression and other personality disorders. Stress has also been made worse by the COVID-19 pandemic’s effects on people’s lives [6]. These mental illnesses are in desperate need of mental health care; however, there is a treatment gap ranging from 74% to 90% for such services. 

There are several approaches to record/collect the human stress levels. A phonocardiography (PCG) signal can be obtained using an electronic stethoscope, which can be utilized as a valuable diagnostic tool in rural locations, with babies, and for homecare purposes [5]. Electroencephalography (EEG), electrooculography (EOG), electromyography (EMG) and electrocardiography (ECG) are the four methods that are utilized most frequently for the purpose of recording physiological signals in response to generated approaches. Photoplethysmography (PPG) also plays a critical role in the collection of physiological signals [7]. Conferring with the deferent literature, bio-chemical and bio-logical-based methodologies have produced contradictory results; these were attributed to hormone instability. 

Mental stress is an important belief that is slowly gaining attention in different research fields related to neuroscience, psychology, medicine and other fields such as sentiment computing. As a result, it is critical to investigate stress using a variety of methods, such as EEG data. Signals such as EEGs are extremely effective at revealing correlations between various rhythmic signals [8]. Due to the scarcity of professional automation and semi-automation, the study of different multimodal signals such as EEGs/ECGs is critical [8]. Big data and the Internet of Medical Things have made it more important than ever to diagnose, detect, and treat mental illnesses [9,10]. Consequently, more than 45 percent of high school scholars are stressed, which has an undesirable impact on their learning performance [11,12]. Stress, on the other hand, manifests itself in a variety of ways [13]. As a consequence of this, it is absolutely necessary to research the effects of stress, utilizing individual EEG signals and the data on the human brain’s bioelectrical transmissions [14,15]. Based on the EEG data obtained from the scalp, it is possible to examine the electrical activities of the brain [16,17]. Additionally, electroencephalography has developed into a crucial non-invasive method for gauging brain activity that can detect anomalies, abnormalities, and mental illnesses [18,19]. The EEG signal has been widely utilized to identify and analyze human stress [20], particularly in the frontal lobe [21]. Many recent studies have focused on using EEG signals to detect and diagnose mental stress, as well as the link between frontal lobe EEG alpha-bands and emotional states activity [22,23].

Generally, EEG signals are typically electrical recordings that are random, non-stationary, non-correlated and non-linear in character. Therefore, the proper diagnosis of disease from EEG signals requires advanced signal processing tools to identify the brain’s rhythms [24]. There are four types of features of extraction techniques for raw EEG signals: (i) time-domain-based, (ii) frequency domain-based, (iii) time–frequency domain-based, and (iv) spatial-time–frequency domain-based [24]. Table 1 demonstrates the feature extraction methods for classification procedures. Frequency and temporal investigations can improve EEG studies [4]. This research applied a time–frequency analysis technique for EEG signals called discrete wavelet transform (DWT) analysis. The DWT technique is useful because it can convert fluctuating EEG signals into more stable linear ones [25,26,27].

### 1.1. Machine Learning/Deep Learning for Classification of EEG Signal

After successful extraction of the features from an EEG signal, machine learning (ML)/deep learning (DL) models are used to classify the EEG Signals. ML/DL models can be able to learn automatically from data without any human interaction [28,29,30,31,32,33,34,35,36].

Recently, DL techniques have increasingly been used for the analysis of EEG signals, due to their remarkable characteristics [34]. A DL model can be an artificial neural network (ANN) with multiple hidden layers, such as CNN, RNN, etc.. DL models use multiple layers of neural connections for extracting different features from the data and progressively improving the accuracy of the results. These models showed efficient results in EEG signals classification for several disease diagnoses [27,34,36,37]. In addition, DL algorithms perform better than typical ML models when it comes to the classification of EEG data (brain signals) [24]. Table 1 shows the approaches of previous research in the analysis of EEG signals.

The previous research used various ML approaches, such as naïve Bayes (NB) and support vector machine (SVM), to detect and classify stress conditions using EEG signals [38], and proposed different stress classification techniques. The improved Elman neural network (IENN) was used to develop a stress detection system in [39]. A cognitive design of an autonomously intelligent agent implemented an ANN [40]. 

Studies of EEG signals routinely employ a wide variety of ML models, including SVM, neural networks (NN), k-nearest neighbor (KNN), stochastic gradient decent (SGD) and linear regression (LR), multilayer perceptron (MLP), random forest (RF) and fuzzy logic (FL) [41,42,43]. 

**Table 1:** Research analysis of previous models using ML and DL.

Classifier	Volunteers/Subjects	Feature Engineering (Domain)	Pros. of Classifier	Cons. of Classifier	Accuracy
SVM	15 volunteers [41]	Correlation analysis (Time)	Works effectively when classes are well-separated	Unsuitable for large data sets	86.94%
MLP	33 subjects, eyes open and closed conditions [44]	Neuro-physiological Features (Time)	More efficient on non-linear data	Classification task computation are complex and consuming time	85.20%
SVM	6 subjects’ EEG dataset [45]	Hilbert Huang Transform (Time-Frequency)	-	Performs poorly when target classes overlap due to noise.	89.07%
LR	4 EEG channels features of 27 subjects [38]	Band power (Frequency)	Works well when data are linearly separable.	Requires average or no independent variable multi-collinearity	98.76%
SVM	17 patients were taken from subjects [46]	NIL	-	-	90.0%
SVM	34 patients were taken from subjects [47]	Band Power (Frequency)	-	Needs extensive testing such as cross validation	85.0%
NB	48 practice patterns were taken from subjects [48]	DWT (Time and frequency)	Process high-dimensional data efficiently	NB struggles to predict minorities class data	91.60%
DL Network	32 samples were taken from the subjects [49]	Power spectrum density (Frequency)	The model learns relevant features without manual feature engineering.	The training data, and model’s performance can decline in diverse hardware resources.	53.42%
RF	17 scalp patients and 10 intracranial were taken from subjects [50]	DWT (Time and frequency)	It automatically selects a subset of characteristics at each split, reducing the causes of dimensionality and irrelevant features.	Low-cardinality features may be less important or require preprocessing to avoid bias.	62.00%
FL	19 patients were taken from subjects [51]	Band Power (Frequency)	It permits the formulation of rules that account for varying degrees of uncertainty and exceptions	Complex fuzzy systems demand more processing and memory, making them unsuitable for real-time or resource-constrained applications.	91.80%
KNN	32 healthy subjects only [52]	DWT (Time and frequency)	It detects linear and non-linear data relationships	Struggles with class imbalances	95.69%
Long Short-Term Memory (LSTM)	32 EEG channels of 32 subjects [53]	Band power (Frequency)	Successfully captures long-term relationships and can alleviate the vanishing gradient issue that is typical in standard RNNs, making training and optimization simpler.	It is susceptible to overfitting, especially when the model has a high number of parameters and the training data are restricted.	94.69%
(Bidirectional Long Short-Term Memory) BiLSTM-LSTM	14 EEG channels of 48 subjects [54]	Power spectral density (Frequency)	Processes data in both directions, and is able to properly capture past and future contexts of the data	Requires an extensive amount of data to train successfully, this may pose a problem if there are not enough labelled data.	97.80%
LR, NN, RNN	1488 abnormal, 1529 normal patients were taken from subjects [55]	Raw EEG	RNNs can learn context from previous inputs	RNNs face vanishing gradient problem	RNN achieve3.47% more
VGG16-CNN	16 and 19 channels were taken from 45 and 28 subjects, respectively [35]	Continuous Wavelet Transform (Time-Frequency)	VGG16 can be used as a feature extractor or as a starting point for transfer learning	VGG16 requires more computation compared other more streamlined CNN architectures such as ResNet or Inception.	98%
2D-CNN-LSTM	5 channels are taken from 60 subjects [36]	Raw EEG	In hybrid model, CNNs are powerful in automatically learning hierarchical features from input data and LSTM networks; on the other hand, can handle temporal variations and long-term dependencies in sequential data.	It is susceptible to overfitting, especially when the model has a high number of parameters and the training data are restricted.	72.55%
CNN + LSTM	60 channels features of brain EEGs were taken from 54 subjects [27]	Fuzzy Entropy and fast Fourier transform (Frequency)	Hybrid DL model performed well on high dimension data	Hybrid DL Model takes long computation time for training	99.22%

**Figure 1:**
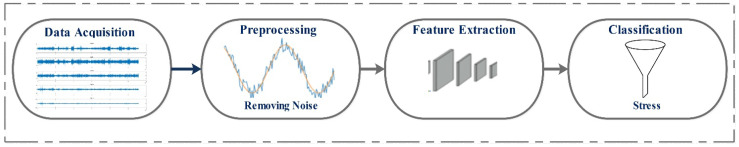
EEG signal analysis general steps.

Figure 1 shows how researchers collect, analyze, and classify brain signals using EEG signal analysis. The steps are as follows: data capture, preprocessing, feature extraction and categorization [56].

### 1.2. Research Contributions

This study presents a novel hybrid deep learning approach for stress detection. The simultaneous task EEG workload (STEW) dataset was used [57], and an effective technique called DWT for frequency band decompression and noise removal from raw EEG signals was utilized. DWT delivers reliable frequency and timing information at low and high frequencies. Hence, the DWT is ideal for asymmetrical data analysis [58,59]. Decomposed EEG signals are taken as the input to a CNN-based automatic feature selection technique. For the classification of stress levels, a hybrid combination of deep learning models called LSTM, BiLSTM, two layers of Gated Recurrent Unit (GRU) and RNN were applied to the classification of human stress. The hybrid combination of these techniques provided improved performance, and could efficiently handle long-term dependencies in non-linear brain signals. The proposed hybrid DL model, in contrast to more traditional methods of anomaly identification, attained efficient accuracy.

## 2. Approaches and Data Description

This section describes the EEG dataset used. It introduces DWT, which was used for signal de-noising and decomposing; CNN, which was used for automatic feature extraction; and RNN, LSTM, BILSTM and GRU, which are briefly introduced.

### 2.1. Dataset Descriptions

**Figure 2:**
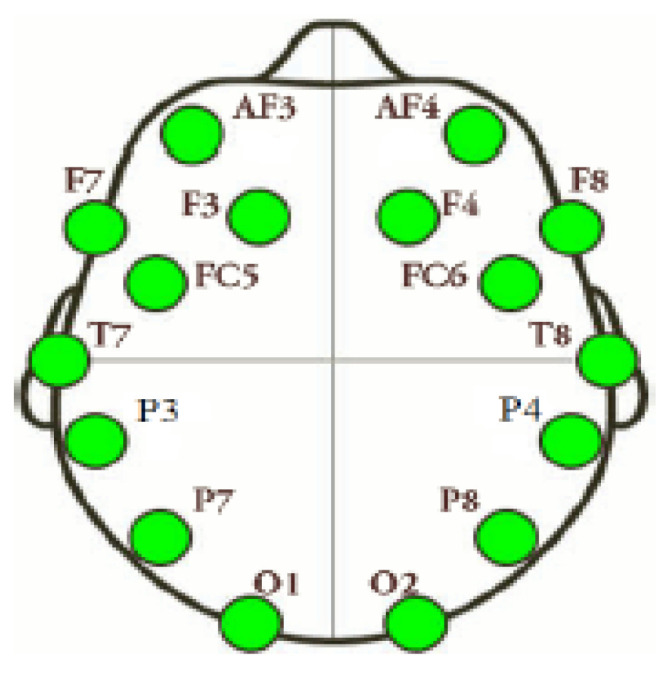
Positions of electrodes according to the 10–20 international system.

In this research, a STEW, simultaneous task EEG workload [57] dataset, was used. This dataset consisted of total 48 subjects. A commercial psychological test single-session simultaneous capacity (SIMKAP) experiment was performed, and the EEG signal activity was evaluated with MATLAB EEGLAB toolbox [60]. An emotive EPOC (high resolution, multi-channel, wireless neuroheadset) EEG device was used for EEG data collection, with 128 Hz as the sampling frequency. According to the 10–20 international system, the device had fourteen electrodes located at AF3, F3, F7, FC5, T7, P7, O2, O1, T8, P8, FC6, AF4, F4 and F8. This research considered only a SIMKAP experiment based on subjects’ ratings on a scale of 1–9. In reality, the inspection process as a whole was a form of validation for the participant, who faced a greater burden while performing the test. A 1 to 3 rating was a low burden, 4 to 6 was a moderate burden and 7 to 9 was a high burden. The EEG recordings consisted of a total of fourteen channels. Figure 2 depicts the positions of the electrodes, according to the 10–20 international system.

Before starting any analysis, it was critical to process the raw EEG signal data to remove the artefacts that were caused by muscle movement, and to clear the data of noise. More details of the dataset description are presented in [57].

The general steps were as follows:

(a) Apply a 1 Hz high-pass filter on the raw data.
(b) Eliminate the line noise.
(c) Carry out artifact subspace reconstruction (ASR).
(d) Re-assign data to the average.

### 2.2. Discrete Wavelet Transform

The wavelength techniques were already fulfilled the signal decomposing along with de-noising significantly. The transformation coefficients could be estimated to the initial signal [58]. Wavelets can be used to classify the neighborhood features of the signals in both the frequency and time domains. The frequency domain generates low-frequency wavelets to compare to the large-scale time domain [61]. The continuous wavelet transform (CWT) of signal x(n) is enumerated as follows:

(1) $${\mathrm{WT}}_{x}\left(a,\tau \right)=\frac{1}{\sqrt{a}}\int_{-\infty }^{\infty }\;x\left(n\right)\psi \left(\frac{n-\tau }{a}\right)\delta n$$

where a is the scale displacement, τ is the time displacement, and ψ(i) indicates a wavelet basis function. EEG signals are discrete signals; for this reason, DWTs are essential requirements of discrete wavelets. In comparison to the CWT, the DWT restricts the a and τ from the wavelet basis function ψ(a,τ) to different discrete points, i.e., the scale and displacement are discretized. The discrete wavelet basis function is expressed as ψ(j,l) (n) = 2^(−i/(2)) ψ(2^(−j) n−l) where, j ∈ Z, l ∈ Z indicates DWT.

(2) $${\mathrm{WT}}_{x}\left(j,l\right)=\int x\left(n\right)\psi^{*}\left(n\right)\delta t$$

**Figure 3:**
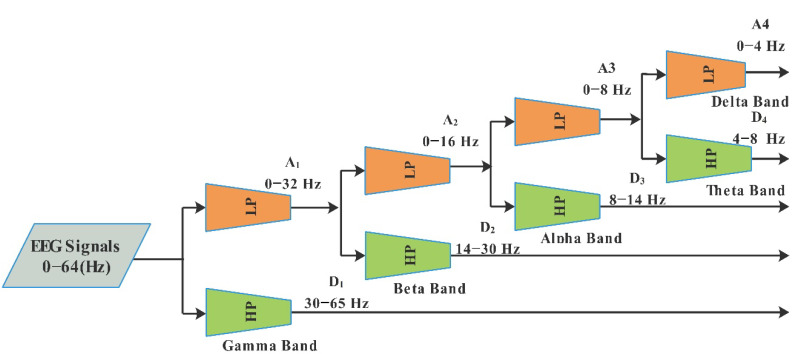
Discrete wavelet transforms analysis.

In DWT, the scaling function brings off both the low- as well as the high-pass filter. The procedure of the DWT workflow is shown in Figure 3, where approximation coefficients have a low-pass frequency resolution but a high time resolution, whereas the detail coefficient has the reverse condition [61]. The wavelets of the four-level EEG signals are decomposed using low-pass (LP) and high-pass (HP) filter coefficients (Figure 3), which are detail coefficients (D1, 30–65 Hz; D2, 14–30 Hz; D3; 8–14 Hz; D4; 4–8 Hz, Theta) and approximation coefficients (A1: 0–32 Hz; A2: 0–16 Hz; A3: 0–8 Hz; A4: 0–4 Hz, Delta) [9].

### 2.3. Convolutional Neural Network (CNN)

The CNN structure can mimic the activity of the human brain’s composite cerebral cortex. To train a multiplex model, it predicts based on a large training dataset that uses many algorithms, such as back propagation and gradient descent optimization, to find out effective features. It utilizes a multiple number of filtering techniques, non-linear activation and normalization methods to extract different important features [62,63].

The recommended simple CNN input layer is involved by the convolutional layer, which then passes the result to the next layer. The filter application and the feature extraction properties in the convolutional layers act as an input signal [64]. Each sub-sample input layer minimizes its dimension to reduce different number of parameters. It learns how to reduce calculation costs by utilizing standard discretization max-pooling-1D blocks. The flattened layer is utilized for traditional multidimensional data to flatten out. In the classification process, dropout layers prevent the loss of validity by normalizing and improving the neural network over-fitting problem.

### 2.4. Recurrent Neural Networks (RNN)

RNN are powerful and robust in nature, and consist of recurrent networks along with internal memory. Since RNN weights are considered for both the input and looping back output signals, these types of weights are adjusted with the help of gradient descent or back propagation [64] algorithm. The deficit in RNN is long-term dependencies [65], while the LSTM can solve this problem due to the design of its repeating module. RNN are time delay networks with training complexity issues, which is a key problem, because at each back propagation step of computation there is gradient loss. Therefore, the LSTM model may be utilized in place of the RNN model without having these side effects [66].

### 2.5. Long Short-Term Memory (LSTM)

**Figure 4:**
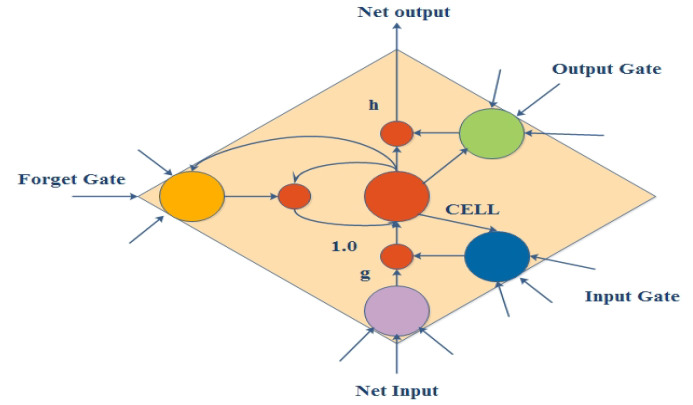
Structure of an LSTM memory cell.

The LSTM network presents a unique configuration known as a memory cell [64,67]. This memory cell consists of four major components: a neuron, a forget gate, an input gate, and an output gate with a self-recurrent structure (Figure 4). The ability of cells to store and access information for longer durations is supported by these gates.

The hidden states are calculated by the LSTM network using the following equations:

(3) $$i=\sigma \left(x_{n}U^{i}+s_{n-1}W^{i}\right)$$

(4) $$f=\sigma \left(x_{n}U^{f}+s_{n-1}W^{f}\right)$$

(5) $$o=\sigma \left(x_{n}U^{\circ }+s_{n-1}W^{\circ }\right)$$

(6) $$g=\tanh\left(x_{n}U^{g}+s_{n-1}W^{g}\right)$$

(7) $$c_{n}=c_{n-1}\circ f+g\circ i$$

(8) $$s_{n}=\tanh\left(c_{n}\right)\circ o$$

(9) $$y=\mathrm{softmax}\left({\mathrm{Vs}}_{n}\right)$$

Traditionally, neural networks are used, such as feedforward neural networks as well as recurrent neural networks. In essence, a feedforward neural network is an ANN in which the output of any layer does not affect the overall performance of that same layer, i.e., there is no cycle formed by the connections between the two units. However, feedforward networks are processed to the network by both the input and output layers.

### 2.6. Bidirectional LSTM

**Figure 5:**
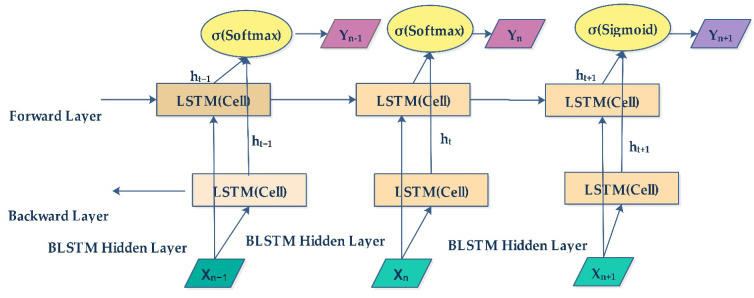
BiLSTM model architecture.

The BiLSTM learning technique is a series of processing models that includes two LSTM networks: the first one acts in a forward direction, and second one in a backwards direction [68,69]. BiLSTMs effectively increase the amount of information available to the network, giving the algorithm more context. Figure 4 shows a BiLSTM model architecture which consists of forward, backward and hidden layers. In the BiLSTM model (Figure 5) architecture, σ is the activation function for the layers, and x and y are the input and output, respectively.

### 2.7. Gated Recurrent Units (GRU)

**Figure 6:**
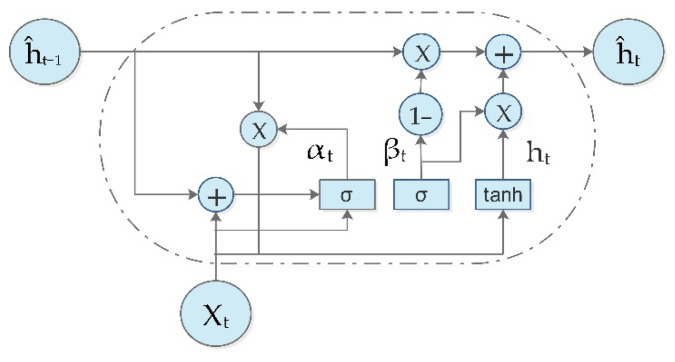
Structure of a GRU cell.

Traditional RNN architectures suffer from vanishing and rising gradients [70]; this makes optimization challenging, and prevents the networks from learning long-term dependencies. To address this issue, several RNN modifications have been proposed, the most popular of which are long short-term memory units (LSTMs) [71,72]. In comparison to LSTMs, GRUs are easier to implement, require fewer parameters, and have better performance in a number of scenarios [73]. Figure 6 shows the structure of a GRU cell

Where reset $\left(\alpha_{t}\right),$ update $\left(\beta_{t}\right)$ and output $\left(h_{t}\right)$ are gates at time t. outputs of gates$\;{ĥ}_{t}\;$and ${ĥ}_{t-1}$ are the output at times t and t − 1, respectively, Input$\;(X_{t})\;$is at time t, and the activation functions (σ, tanh). $W_{\alpha },{\;W}_{\beta },{\;W}_{ĥ}{\;\mathrm{and}\;W}_{o}$ are the weights for the input and output of the models.${\hat{y}}_{t}\;$shows the output of the training sample at time t. The calculation process of the memory unit is expressed by Equations (10)–(14).

(10) $$\alpha_{t}=\sigma \left(W_{\alpha }\cdot \left[{ĥ}_{t-1},X_{t}\right]\right)$$

(11) $$\beta_{t}=\sigma \left(W_{\beta }\cdot \left[{ĥ}_{t-1},X_{t}\right]\right)$$

(12) $$h_{t}=\tan h\left(W_{ĥ}\cdot \left[\alpha_{t}*{ĥ}_{t-1},X_{t}\right]\right)$$

(13) $${ĥ}_{t}=\left(1-\beta_{t}\right)*{ĥ}_{t-1}+z_{t}{*h}_{t}$$

(14) $${ŷ}_{t}=\sigma \left(W_{o}\cdot {ĥ}_{t}\right)$$

## 3. The DWT-Based Hybrid DL Models

This section describes the proposed methods with a flow diagram and the parameter settings. These experiments were performed using a Python rich library tool [74], using a hardware configuration of RAM-32GB, i9 and CUDA-GPU. The following describes the steps of the proposed methodology.

Step 1: Feature extraction from EEG signals

**Figure 7:**
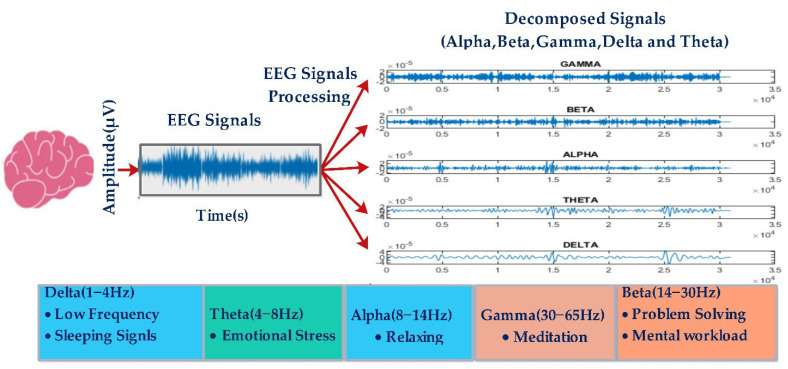
Decomposed EEG signal.

The DWT is a familiar tool used to remove noise, as well as for feature extraction from the signals. The wavelet decomposition of a noisy signal emphasizes essential signal information in a few large absolute-valued wavelet coefficients, without changing the random noise distribution [75]. In this research, each subject has fourteen EEG channels. The signal is extracted using DWT from the EEG dataset, and signals are decomposed in four levels with Daubechies (dB4) wavelet function. Order 4 of the Daubechies wavelet function performs better than the other orders [76]. The de-noising method is used with a free distributed hypothesis test threshold (FDR), tunable oscillatory behavior (Q-factor) with a value of 0.05; the threshold rule is hard and independent of the noise level. Figure 7 shows a decomposed signal in frequencies called Alpha, Beta, Gamma, Delta and Theta.

Step 2: CNN and max pooling layer description

The CNN selects features from decomposed EEG signals in the model. In signal processing, the CNN, a deep learning subset, has gained attention [77]. In the model, one CNN-1d layer is continuously used with filter size ‘128′, kernel size 1, padding set as ‘valid’, and activation set as ‘Softmax’. These filters were used as inputs to the next layer. For the purpose of feature extraction from input signals, the ‘Softmax’ activation function was employed to describe a probability distribution over an n-valued discrete signal with kernel size 1. For the input signals to be entirely covered by the filter, it was assumed that all dimensions are accurate. The CNN layer receives pre-processed EEG signals as the input, and the max-pooling filter acts as a window through which only the highest score is selected for the output. The hit-and-trial method was used to select all of the learning parameters.

Step 3: Different hybrid combinations for stress classification

**Figure 8:**
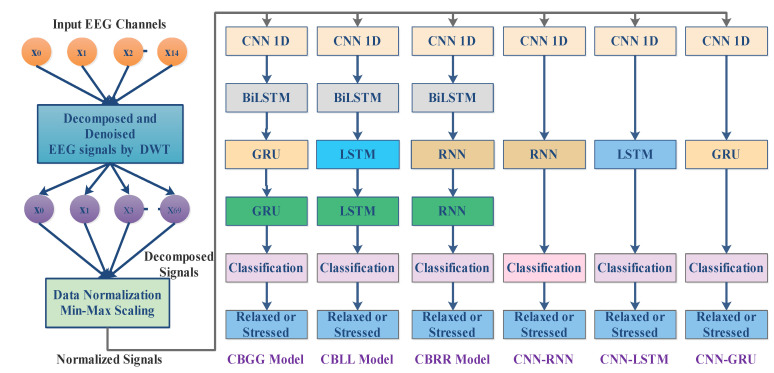
Combination DWT-based hybrid DL models.

In this research, six hybrid combinations with the BiLSTM, RNN, LSTM and GRU models were developed for classifying human stress levels. The hybrid combination of DL models may provide improved performance, and can handle long-term dependencies in non-linear brain signals. The hybridization of DLs leverages the strengths of different DL models, and removes the limitations of single DL models. It enhances the performance efficiency for EEG signal classification tasks [27,35,36]. Figure 8 shows the six hybrid model combinations. The described CNN layers functioning in step 2 were same for all combinations. In each of the first three combinations (Figure 8), the BiLSTM layer was followed by two GRU layers (CBGG) or two LSTM layers (CBLL), or two RNN layers (CBRR). Later, three were CNN–RNN, CNN–LSTM and CNN–GRU. Table 2 represents each hybrid model’s parameter description. In order to solve the issue of over-fitting that arises during the learning process, a dropout unit rate was set as 0.2. A single neuron with a sigmoid activation function was employed to feed information into the last dense layer. The hit-and-trial method was utilized throughout the entire process of configuring the parameters. The model’s objective function was set as “binary_crossentropy” and the optimizer was “Adam”. The other fitting parameters are epoch (200) and batch_size (50) were chosen based on the hit-and-trial approach.

## 4. Results and Analysis

This section presents findings based on the matrices, convergence and receiver operating characteristic (ROC) curves, which are provided for a visual comparison of the hybrid models.

### 4.1. Metrics-Based Performance

In this study, the data were classified into two classes, the stress state and the relaxed state. The binary classification was evaluated on the basis of confusion matrix parameters. The parameters’ names and formulations are specified in Table 3.

### 4.2. Performance Evaluation

The performance of the proposed hybrid DWT-based CBGG model was compared with the CBRR, CBLL, CNN–RNN, CNN–LSTM and CNN–GRU models to prove its better efficiency. The STEW [48] dataset was used to prove the usability of the hybrid models. The dataset had 14 channels and 921,600 (128 sampling frequency × 150 s recoding time × 48 participants) data points per channel, with 70 percent used for instruction and 30 percent used for testing. The training data were likewise divided 70:30 for the purposes of validating and testing the performance of the models.

**Figure 9:**
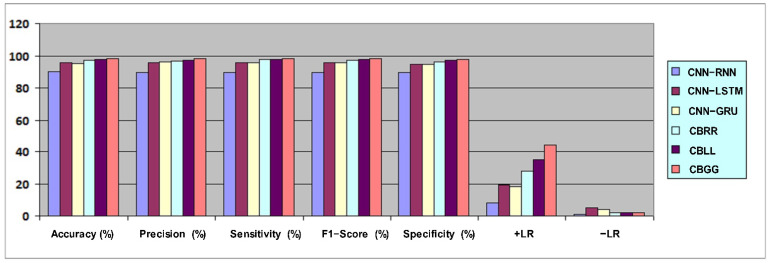
Graphical comparison of the proposed model with other models.

The proposed CBGG model’s outcomes were evaluated using the following metrics: accuracy, sensitivity, F-score, specificity, precision, +LR and −LR. Table 4 represents a comparison of the models based on stated parameters. The proposed model outperformed the existing models, and achieved highest accuracy of 98.10%. The CBRR and CBLL models showed better efficiency compared to the CNN–RNN, CNN–LSTM and CNN–GRU hybrid models. Furthermore, the performance metrics scores for the sensitivity (98.08%), F-score (98.20%), specificity (97.76%), precision (98.27%), +LR (44) and −LR (0.02) of the proposed CBGG model were higher compared to those of the other models. Figure 9 shows a graphical representation of confusion matrix parameters over the proposed hybrid model in comparison with the other models.

### 4.3. Convergence Curve Analysis

**Figure 10:**
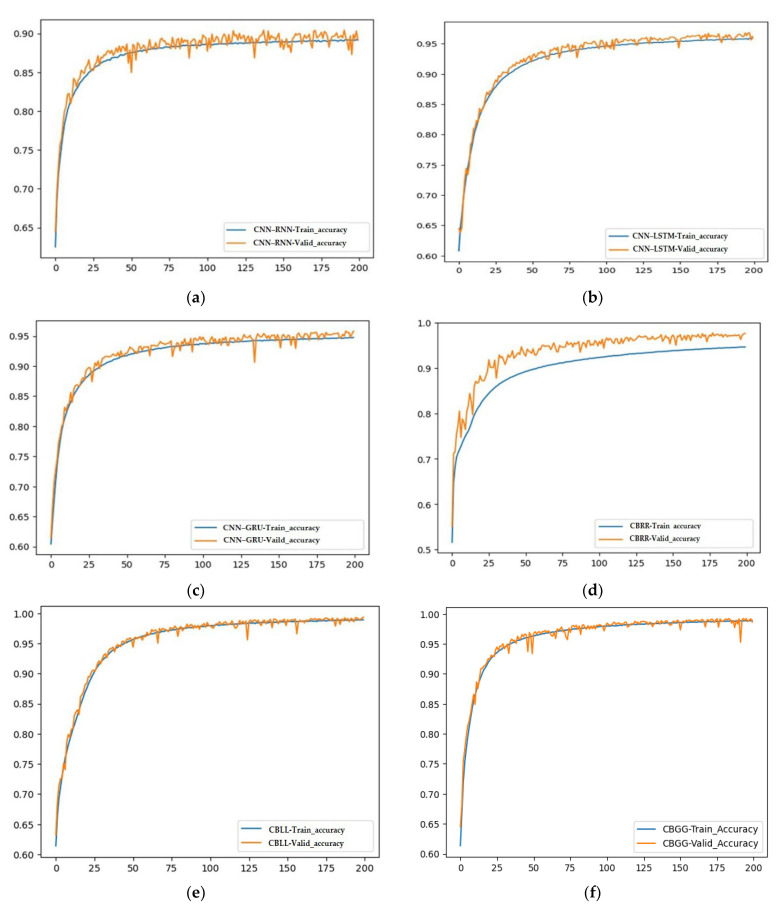
Models train vs. validation accuracy convergence curve (**a**) CNN–RNN (**b**) CNN–LSTM, (**c**) CNN–GRU, (**d**) CBRR, (**e**) CBLL, (**f**) CBGG.

A convergence curve for both the training and validation phases can be thought of as a representation of the optimum possible value for a learning parameter in relation to the accuracy, with regard to the loss function. Figure 10a–f show the training accuracy curve and validation accuracy curve traces for the hybrid CNN–RNN, CNN–LSTM, CNN–GRU, CBRR, CBLL and CBGG models, respectively (200 epochs). The proposed model’s (CBGG) (Figure 10f) train and validation accuracy showed a faster convergence rate compared to that of the other developed models, with a higher accuracy of 98.10%.

### 4.4. Receiver Operating Characteristic (ROC) Curve Analysis of Models

**Figure 11:**
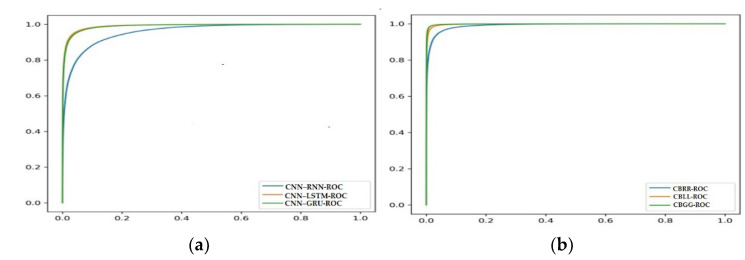
(**a**) ROC curves of the CNN–RNN, CNN–LSTM, CNN–GRU and (**b**) CBRR, CBLL and CBGG models.

The ROC curve is a measurement of how well a model distinguishes between the stressed and relaxed states. Since there were so many inconsistent data points in the collection, the performance indicator based on confusion was insufficient. It was possible to determine the ratio of accurate positives to erroneous ones using an ROC curve, which was applied to the test data. On the *X*-axis, the false positive rate was plotted, while the real positive rate was plotted on the *Y*-axis. Figure 11a shows ROC curves of the CNN–RNN, CNN–LSTM and CNN–GRU models, and Figure 11b shows the ROC curves for the CBRR, CBLL and CBGG models. In Figure 11b, the ROC show better efficiencies for the CBRR, CBLL and CBGG models compared to the other three hybrid models in Figure 11a. The proposed model (CBGG), which includes a larger ROC covered area (area under curve), indicates that it performed better than the CBRR and CBLL models.

### 4.5. Comparison of Proposed and Existing Works

This section compares the proposed research with existing machine learning models built using EEG channels and feature selection methods on the same dataset. The detection of relaxation and tension from EEG signals has also been the subject of numerous studies in the literature. Table 5 shows a comparison of the CBGG’s performance with those of existing models.

### 4.6. Validation of Proposed Model

To provide quantitative statistical results, predictive models were constructed on a dataset and then evaluated using resampling techniques. Lastly, statistical analysis was carried out to select the ideal model. The average classification accuracy in this study, based on training and testing the CBGG model using a stratified 10-fold cross-validation method, was 97.60%.

### 4.7. Limitations of the Proposed Hybrid DL Models

The main limitation of DL-based hybrid models is the complexity due to significant parameter tuning, which required higher running time. Furthermore, the selection of different hybrid combinations was a tedious task in proving the validity of the models on the EEG dataset.

## 5. Conclusions

This study developed DWT-based hybrid deep learning models used for the classification of stress using a STEW dataset that consisted of a total of 48 subjects. The occurrence of stress is quite common, and it causes many health-related issues, such as insomnia, decreased immunity, infections, cervical impairments and migraines. EEG signals are one of the reliable tools for stress detection. Therefore, stress can be cured before it becomes worse. In this research, an EEG signal is used as the input, and five different frequency bands were extracted using a DWT. After frequency extraction, a CNN was used for automatic feature extraction to achieve better prediction performance. Finally, a deep hybrid model named as CBGG showed significant performance for stress level detection. The attained accuracy using CBGG was 98.10%. The attained results demonstrate the feasibility of using EEG signals for the detection of stress. Therefore, this method is appropriate for the clinical intervention and prevention of mental and physical problems. In future research, the proposed automatic feature extraction-based hybrid deep learning model will be tested in prediction tasks that involve a greater number of EEG datasets. Furthermore, the large number of parameters of these hybrid combinations can be reduced, in order to run them in edge devices.

## Figures and Tables

**Table 2:** Model parameter descriptions.

Model Sequential Layers	Layer	Parameters and Values
CBRR	CNN-ID MaxPooling1D BiLSTM Layer RNN Layer RNN Layer Dropout Total Parameters	Filter = 128, kernel size = 1, padding = valid, activation = softmax Pool_size = 1 Filter =64 Filter =32 Filter =16 0.2 112,927
CBLL	CNN-ID Layer MaxPooling1D BiLSTM Layer LSTM Layer LSTM Layer Dropout Total Parameters	Filter = 128, kernel size = 1, padding = valid, activation =softmax Pool_size = 1 Filter = 64 Filter =32 Fiter =16 0.2 153,681
CBGG	CNN-ID Layer MaxPooling1D BiLSTM Layer GRULayer GRU Layer Dropout Total Parameters	Filter = 128, kernel size = 1, padding = valid, activation =softmax Pool_size = 1 Filter = 64 Filter =32 Fiter =16 0.2 117,657
CNN-RNN	CNN-ID Layer MaxPooling1D RNN Dropout Total Parameters	Filter = 128, kernel size = 1, padding = valid, activation =softmax Pool_size = 1 Filter = 64 0.2 12,673
CNN-LSTM	CNN-ID Layer MaxPooling1D LSTM Dropout Total Parameters	Filter = 128, kernel size = 1, padding = valid, activation =softmax Pool_size = 1 Filter = 64 0.2 49,729
CNN-GRU	CNN-ID Layer MaxPooling1D GRU Dropout Total Parameters	Filter = 128, kernel size = 1, padding = valid, activation =softmax Pool_size = 1 Filter = 64 0.237,569

**Table 3:** Metrics formulation of parameters.

Parameter	Formula
Precision	$\frac{TP}{TP+FP}*100$
Sensitivity	$\frac{TP}{TP+FN}*100$
Specificity	$\frac{TN}{FP+TN}*100$
F1-Score	$\frac{2\;*\;(PPV\;*\;\mathrm{Sensitivity})\;}{PPV+\;\mathrm{Sensitivity}\;}$
Accuracy	$\frac{TP+TN}{TP+FN+FN+TN}\;*\;100$
Positive Likelihood Ratio (+LR)	$\frac{\;\mathrm{Sensitivity}\;}{100-\;\mathrm{Specificity}\;}$
Negative Likelihood Ratio (−LR)	$\frac{100-\;\mathrm{Sensitivity}\;}{\;\mathrm{Specificity}\;}$

**Table 4:** Model comparison results.

Model	Accuracy (%)	Precision (%)	Sensitivity (%)	F1-Score (%)	Specificity (%)	+LR	−LR
CNN–RNN	89.91	89.70	89.83	89.80	89.80	8	0.12
CNN–LSTM	95.60	95.90	95.64	95.90	94.90	19	0.05
CNN–GRU	95.20	96.09	95.77	95.50	94.70	18	0.04
CBRR	97.10	96.53	97.74	97.14	96.45	28	0.02
CBLL	97.54	97.32	97.80	97.56	97.27	35	0.02
**CBGG**	**98.10**	**98.27**	**98.08**	**98.20**	**97.76**	**44**	**0.02**

**Table 5:** Comparison with the most relevant models in dealing with the identification of stress in STEW EEG signals.

Feature Extraction/ Selection	Classifier	Accuracy	Cross-Validation
Particle Swarm Optimization (PSO) [46]	BiLSTM, LSTM	86.33%	-
PSD features via FFT [48])	KNN, SVM	69.00%	-
CNN-based features from DWT signals	CBGG	98.10%	97.60% (Stratified 10-Fold)

## Data Availability

Publicly available dataset [49].

## References

[1] Sharma R., Chopra K. (2020). EEG signal analysis and detection of stress using classification techniques. J. Inf. Optim. Sci..

[2] Mikhno I., Koval V., Ternavskyi A. (2020). Strategic management of healthcare institution development of the national medical services market. ACCESS Access Sci. Bus. Innov. Digit. Econ..

[3] Qadri A., Yan H. (2023). To promote entrepreneurship: Factors that influence the success of women entrepreneurs in Pakistan. Access J..

[4] Singh S.K., Singh S.S., Singh V.L. (2023). Predicting adoption of next generation digital technology utilizing the adoption-diffusion model fit: The case of mobile payments interface in an emerging economy. Access J..

[5] Cheema A., Singh M. (2019). Psychological stress detection using phonocardiography signal: An empirical mode decomposition approach. Biomed. Signal Process. Control..

[6] Petrova M., Tairov I. (2022). Solutions to Manage Smart Cities’ Risks in Times of Pandemic Crisis. Risks.

[7] Salankar N., Koundal D., Qaisar S.M. (2021). Stress classification by multimodal physiological signals using variational mode decomposition and machine learning. J. Health Eng..

[8] AlShorman O., Masadeh M., Bin Heyat B., Akhtar F., Almahasneh H., Ashraf G., Alexiou A. (2022). Frontal lobe real-time EEG analysis using machine learning techniques for mental stress detection. J. Integr. Neurosci..

[9] Hasan M.J., Kim J.M. (2019). A hybrid feature pool-based emotional stress state detection algorithm using EEG signals. Brain Sci..

[10] AlShorman O., Alshorman B., Alkahtani F. (2021). A review of wearable sensors based monitoring with daily physical activity to manage type 2 diabetes. Int. J. Electr. Comput. Eng..

[11] Dushanova J., Christov M. (2014). The effect of aging on EEG brain oscillations related to sensory and sensorimotor functions. Adv. Med. Sci..

[12] Mason A.E., Adler J.M., Puterman E., Lakmazaheri A., Brucker M., Aschbacher K., Epel E.S. (2019). Stress resilience: Narrative identity may buffer the longitudinal effects of chronic caregiving stress on mental health and telomere shortening. Brain Behav. Immun..

[13] Belleau E.L., Treadway M.T., Pizzagalli D.A. (2019). The Impact of Stress and Major Depressive Disorder on Hippocampal and Medial Prefrontal Cortex Morphology. Biol. Psychiatry.

[14] Fernández J.R., Anishchenko L. (2018). Mental stress detection using bioradar respiratory signals. Biomed. Signal Process. Control..

[15] Heyat M.B., Hasan Y.M., Siddiqui M.M. (2015). EEG signals and wireless transfer of EEG Signals. Int. J. Adv. Res. Comput. Commun. Eng..

[16] Bakhshayesh H., Fitzgibbon S., Janani A.S., Grummett T.S., Pope K. (2019). Detecting synchrony in EEG: A comparative study of functional connectivity measures. Comput. Biol. Med..

[17] Heyat M.B., Siddiqui M.M. (2015). Recording of eegecgemg signal. Int. J. Adv. Res. Comput. Sci. Softw. Eng..

[18] Pal R., Heyat M.B., You Z., Pardhan B., Akhtar F., Abbas S.J., Guragai B., Acharya K. Effect of Maha Mrityunjaya HYMN recitation on human brain for the analysis of single EEG channel C4-A1 using machine learning classifiers on yoga practitioner. Proceedings of the 2020 17th International Computer Conference on Wavelet Active Media Technology and Information Processing (ICCWAMTIP).

[19] Cea-Canas B., Gomez-Pilar J., Nunez P., Rodriguez-Vazquez E., de Uribe N., Diez A., Perez-Escudero A., Molina V. (2020). Connectivity strength of the EEG functional network in schizophrenia and bipolar disorder. Prog. Neuro-Psychopharmacol. Biol. Psychiatry.

[20] Dushanova J.A., Tsokov S.A. (2020). Small-world EEG network analysis of functional connectivity in developmental dyslexia after visual training intervention. J. Integr. Neurosci..

[21] Olson E.A., Cui J., Fukunaga R., Nickerson L.D., Rauch S.L., Rosso I.M. (2017). Disruption of white matter structural integrity and connectivity in posttraumatic stress disorder: A TBSS and tractography study. Depress. Anxiety.

[22] Zubair M., Yoon C. (2020). Multilevel mental stress detection using ultra-short pulse rate variability series. Biomed. Signal Process. Control..

[23] Goodman R.N., Rietschel J.C., Lo L.-C., Costanzo M.E., Hatfield B.D. (2013). Stress, emotion regulation and cognitive performance: The predictive contributions of trait and state relative frontal EEG alpha asymmetry. Int. J. Psychophysiol..

[24] Luján M.., Jimeno M.V., Sotos J.M., Ricarte J.J., Borja A.L. (2021). A Survey on EEG Signal Processing Techniques and Machine Learning: Applications to the Neurofeedback of Autobiographical Memory Deficits in Schizophrenia. Electronics.

[25] Hosseini M.-P., Hosseini A., Ahi K. (2020). A Review on Machine Learning for EEG Signal Processing in Bioengineering. IEEE Rev. Biomed. Eng..

[26] Aggarwal S., Chugh N. (2022). Review of machine learning techniques for EEG based brain computer interface. Arch. Comput. Methods Eng..

[27] Sun J., Cao R., Zhou M., Hussain W., Bin Wang B., Xue J., Xiang J. (2021). A hybrid deep neural network for classification of schizophrenia using EEG Data. Sci. Rep..

[28] Zuo X.-N. (2020). A machine learning window into brain waves. Neuroscience.

[29] Najafzadeh H., Esmaeili M., Farhang S., Sarbaz Y., Rasta S.H. (2021). Automatic classification of schizophrenia patients using resting-state EEG signals. Phys. Eng. Sci. Med..

[30] Barros C., Silva C.A., Pinheiro A.P. (2021). Advanced EEG-based learning approaches to predict schizophrenia: Promises and pitfalls. Artif. Intell. Med..

[31] Vázquez M.A., Maghsoudi A., Mariño I.P. (2021). An Interpretable Machine Learning Method for the Detection of Schizophrenia Using EEG Signals. Front. Syst. Neurosci..

[32] Mortaga M., Brenner A., Kutafina E. (2021). Towards interpretable machine learning in EEG analysis. German Medical Data Sciences 2021: Digital Medicine: Recognize–Understand–Heal 2021.

[33] da Silva Lourenço C., Tjepkema-Cloostermans M.C., van Putten M.J. (2021). Machine learning for detection of interictal epileptiform discharges. Clin. Neurophysiol..

[34] Gao Z., Dang W., Wang X., Hong X., Hou L., Ma K., Perc M. (2021). Complex networks and deep learning for EEG signal analysis. Cogn. Neurodyn..

[35] Aslan Z., Akin M. (2022). A deep learning approach in automated detection of schizophrenia using scalogram images of EEG signals. Phys. Eng. Sci. Med..

[36] Ahmedt-Aristizabal D., Fernando T., Denman S., Robinson J.E., Sridharan S., Johnston P.J., Laurens K.R., Fookes C. (2020). Identification of Children at Risk of Schizophrenia via Deep Learning and EEG Responses. IEEE J. Biomed. Health Inform..

[37] Nikolaev D., Petrova M. Application of Simple Convolutional Neural Networks in Equity Price Estimation. Proceedings of the 2021 IEEE 8th International Conference on Problems of Infocommunications, Science and Technology (PIC S&T).

[38] Asif A., Majid M., Anwar S.M. (2019). Human stress classification using EEG signals in response to music tracks. Comput. Biol. Med..

[39] Ranjith C., Arunkumar B. (2019). An improved elman neural network based stress detection from EEG signals and reduction of stress using music. Int. J. Eng. Res. Technol..

[40] Dyachenko Y., Nenkov N., Petrova M., Skarga-Bandurova I., Soloviov O. (2018). Approaches to cognitive architecture of autonomous intelligent agent. Biol. Inspired Cogn. Arch..

[41] Betti S., Lova R.M., Rovini E., Acerbi G., Santarelli L., Cabiati M., Del Ry S., Cavallo F. (2017). Evaluation of an Integrated System of Wearable Physiological Sensors for Stress Monitoring in Working Environments by Using Biological Markers. IEEE Trans. Biomed. Eng..

[42] Attallah O. (2020). An Effective Mental Stress State Detection and Evaluation System Using Minimum Number of Frontal Brain Electrodes. Diagnostics.

[43] Ahani A., Wahbeh H., Nezamfar H., Miller M., Erdogmus D., Oken B. (2014). Quantitative change of EEG and respiration signals during mindfulness meditation. J. Neuroeng. Rehabil..

[44] Şeker M., Özbek Y., Yener G., Özerdem M.S. (2021). Complexity of EEG dynamics for early diagnosis of Alzheimer’s disease using permutation entropy neuromarker. Comput. Methods Programs Biomed..

[45] Vanitha V., Krishnan P. (2016). Real time stress detection system based on EEG signals. Biomed. Res..

[46] Aghajani H., Garbey M., Omurtag A. (2017). Measuring mental workload with EEG+ fNIRS. Front. Hum. Neurosci..

[47] Aydin S., Arica N., Ergul E., Tan O. (2015). Classification of obsessive compulsive disorder by EEG complexity and hemispheric dependency measurements. Int. J. Neural Systems.

[48] Amin H.U., Mumtaz W., Subhani A.R., Saad M.N.M., Malik A.S. (2017). Classification of EEG Signals Based on Pattern Recognition Approach. Front. Comput. Neurosci..

[49] Jirayucharoensak S., Pan-Ngum S., Israsena P. (2014). EEG-Based Emotion Recognition Using Deep Learning Network with Principal Component Based Covariate Shift Adaptation. Sci. World J..

[50] Le Douget J.E., Fouad A., Filali M.M., Pyrzowski J., Le Van Quyen M. Surface and intracranial EEG spike detection based on discrete wavelet decomposition and random forest classification. Proceedings of the 2017 39th Annual International Conference of the IEEE Engineering in Medicine and Biology Society (EMBC).

[51] Amezquita-Sanchez J.P., Mammone N., Morabito F.C., Adeli H. (2021). A New dispersion entropy and fuzzy logic system methodology for automated classification of dementia stages using electroencephalograms. Clin. Neurol. Neurosurg..

[52] Li M., Xu H., Liu X., Lu S. (2018). Emotion recognition from multichannel EEG signals using K-nearest neighbor classification. Technol. Health Care.

[53] Nath D., Singh M., Sethia D., Kalra D., Indu S. An efficient approach to eeg-based emotion recognition using lstm network. Proceedings of the 2020 16th IEEE international colloquium on signal processing & its applications (CSPA).

[54] Das Chakladar D., Dey S., Roy P.P., Dogra D.P. (2020). EEG-based mental workload estimation using deep BLSTM-LSTM network and evolutionary algorithm. Biomed. Signal Process. Control..

[55] Roy S., Kiral-Kornek I., Harrer S. (2019). ChronoNet: A deep recurrent neural network for abnormal EEG identification. Proceedings of the Artificial Intelligence in Medicine: 17th Conference on Artificial Intelligence in Medicine, AIME 2019.

[56] Nicolas-Alonso L.F., Gomez-Gil J. (2012). Brain computer interfaces, a review. Sensors.

[57] Lim W.L., Sourina O., Wang L.P. (2018). STEW: Simultaneous task EEG workload data set. IEEE Trans. Neural Syst. Rehabil. Eng..

[58] Lakshmi M.R., Prasad T.V., Prakash D.V. (2014). Survey on EEG signal processing methods. Int. J. Adv. Res. Comput. Sci. Softw. Eng..

[59] Malviya L., Mal S. (2023). CIS feature selection based dynamic ensemble selection model for human stress detection from EEG signals. Clust. Comput..

[60] Ji N., Ma L., Dong H., Zhang X. (2019). EEG Signals Feature Extraction Based on DWT and EMD Combined with Approximate Entropy. Brain Sci..

[61] Aamir M., Pu Y.-F., Rahman Z., Tahir M., Naeem H., Dai Q. (2018). A Framework for Automatic Building Detection from Low-Contrast Satellite Images. Symmetry.

[62] Wu H., Niu Y., Li F., Li Y., Fu B., Shi G., Dong M. (2019). A Parallel Multiscale Filter Bank Convolutional Neural Networks for Motor Imagery EEG Classification. Front. Neurosci..

[63] Mohseni M., Shalchyan V., Jochumsen M., Niazi I.K. (2020). Upper limb complex movements decoding from pre-movement EEG signals using wavelet common spatial patterns. Comput. Methods Programs Biomed..

[64] Smagulova K., James A.P. (2020). Overview of long short-term memory neural networks. Deep Learning Classifiers with Memristive Networks: Theory and Applications.

[65] Bengio Y., Simard P., Frasconi P. (1994). Learning long-term dependencies with gradient descent is difficult. IEEE Trans. Neural Netw..

[66] Kumar D., Singh A., Samui P., Jha R.K. (2019). Forecasting monthly precipitation using sequential modelling. Hydrol. Sci. J..

[67] Hochreiter S., Schmidhuber J. (1997). Long short-term memory. Neural Comput..

[68] Yang J., Huang X., Wu H., Yang X. (2020). EEG-based emotion classification based on Bidirectional Long Short-Term Memory Network. Procedia Comput. Sci..

[69] Malviya L., Mal S. (2022). A novel technique for stress detection from EEG signal using hybrid deep learning model. Neural Comput. Appl..

[70] Abuqaddom I., Mahafzah B.A., Faris H. (2021). Oriented stochastic loss descent algorithm to train very deep multi-layer neural networks without vanishing gradients. Knowl. Based Systems.

[71] Graves A. (2012). Supervised Sequence Labelling. Supervised Sequence Labelling with Recurrent Neural Networks.

[72] Cho K., Van Merriënboer B., Gulcehre C., Bahdanau D., Bougares F., Schwenk H., Bengio Y. (2014). Learning phrase representations using RNN encoder-decoder for statistical machine translation. arXiv.

[73] Chung J., Gulcehre C., Cho K., Bengio Y. (2014). Empirical evaluation of gated recurrent neural networks on sequence modeling. arXiv.

[74] Geldiev E.M., Nenkov N.V., Petrova M.M. (2018). Exercise of machine learning using some python tools and techniques. CBU Int. Conf. Proc..

[75] Faust O., Acharya U.R., Adeli H., Adeli A. (2015). Wavelet-based EEG processing for computer-aided seizure detection and epilepsy diagnosis. Seizure.

[76] Ismail A.R., Asfour S.S. (1999). Discrete wavelet transform: A tool in smoothing kinematic data. J. Biomech..

[77] Acharya U.R., Oh S.L., Hagiwara Y., Tan J.H., Adeli H. (2018). Deep convolutional neural network for the automated detection and diagnosis of seizure using EEG signals. Comput. Biol. Med..

